# Supplementary material for: Assessing the Needs of Those Who Serve the Underserved: A Qualitative Study among US Oncology Clinicians
Source: Cancers (Basel). 2023 Jun 23;15(13):3311. doi: 10.3390/cancers15133311 (PMC10341104; doi:10.3390/cancers15133311)
Supplement: Supplementary file 1 [file cancers-15-03311-s001.zip › cancers-2396974-supplementary.pdf]

Supplemental A: Demographic Pre-Interview Survey

For the purposes of this data collection effort, the American Society of Clinical Oncology Serving the Underserved Task Force is limiting the focus of "underserved" areas and populations to the U.S. Health and Human Services (HHS) definitions. Accordingly, medically underserved areas "have a shortage of primary care health services for residents within a geographic area" and medically underserved populations "are specific sub-groups of people living in a defined geographic area with a shortage of primary care health services." The following are pre-screen and demographic questions to be completed prior to the interview. Please note that data will be reported in aggregate.

1. First and Last Name (Please note this is for internal tracking and will not be reported.)

2. Where does your professional activity primarily occur?
   - Academic medical center/University
   - Physician-owned practice or group
   - Hospital/health-system-owned practice, group, department
   - Training program (student/fellow)
   - Government setting (e.g., Veterans Affairs)
   - Other (please specify)

3. Please provide the number of full-time-equivalent oncology providers (Medical Doctors, Nurse Practitioners, and Physician Assistants) and subspecialists (radiation oncology, surgical oncology, etc.) in your practice.
   - 0-10
   - 11-20
   - 21-30
   - 31-40
   - 41-50
   - >50

4. Do you think that the patient population you provide care for is reflective of the community in which your practice is located?
   - Yes
   - No
   - Not sure
5. To the best of your knowledge, please describe the approximate percentages of your patient population by race/ethnicity (Answers will need to add up to 100%):
   - Asian/Asian American/Native Hawaiian/Pacific Islander
   - Black/African American
   - Hispanic/Latinx
   - Middle Eastern/North African
   - American Indian/Alaska Native
   - Non-Hispanic White
   - Multiracial/ multiethnic
   - A race/ethnicity not listed here

6. To the best of your knowledge, please describe the approximate percentages of your patient population by insurance type. (Answers will need to add up to 100%)
   - Private/commercial
   - Medicare/Medicare Advantage
   - Medicaid
   - Veterans Affairs/other government
   - Other

7. Please estimate the approximate percentage of your clinical time devoted to caring for patients that may be living in poverty, medically uninsured/unable to pay, Medicaid recipients, non-English speaking, or otherwise considered underserved:

- < 25%
- between 25 and 50%
- between 51 and 75%
- < 75%

8. Do you treat pediatric patients that you would characterize as underserved (e.g., living in poverty, medically uninsured/unable to pay, Medicaid recipients, non-English speaking)?
    - Yes
    - No

9. Do you have bilingual personnel at your practice to provide interpretation services for patients?
    - Yes
    - No
    - Not sure
    - I have no non-English-speaking patients

10. Do you reside in the community where you practice?
    - Yes
    - No

11. Do you think of yourself as:
    - Female
    - Male
    - Transgender man/trans man/female-to-male (FTM)
    - Transgender woman/trans woman/male-to-female (MTF)
    - Genderqueer/gender nonconforming/neither exclusively male nor female
    - Additional gender category (or other); please specify: _______________
    - Prefer not to respond

12. What race/ethnicity do you identify as? Select all that apply (can select multiple).
    - Asian American Native Hawaiian or other Pacific Islander
    - Black/African American
    - Hispanic/Latinx
    - Middle Eastern/North African
    - Native American/American Indian
    - Non-Hispanic White
    - Multiracial/Multiethnic
    - A race/ethnicity not listed here, please specify__________
    - Prefer not to respond

13. How many years have passed since the completion of your terminal degree?
    - I am still enrolled in a terminal degree program
    - less than 1 year
    - 1-5 years
    - 6-10 years
    - 11-20 years
    - 20+ years

Supplemental B: In-Depth Interview Guide

Informed Consent and Anonymity

1. PARTICIPANT RIGHTS: Before we begin, I want to make sure you know that this call will be recorded and your participation is voluntary; you can choose not to answer any individual question, or you can end the interview at any time.

2. CONFIDENTIALITY: This is a research conversation intended to improve ASCO's insights into provider unmet needs and practice patterns in providing care for underserved populations. By default, we will keep all the information you provide us confidential here at ASCO. Any report resulting from analysis of the data will be anonymized, unless you would like to choose otherwise. With that said, may I use your name and the name of your practice/institution in the interview report or would you prefer to remain anonymous?

   ☐ ID OK ☐ Prefer anonymity

3. NOTE-TAKING: Finally, in addition to the recording, I want to let you know that the recording will be transcribed and ASCO staff plan to take notes throughout this call to best capture your answers most accurately and in full detail. Ready to begin?

Interview Questions

The participant should guide the conversation loosely through the questions; sub-questions can ensure that each participant answers every aspect of the main questions fully. Unwritten probes should be used in cases where the participant offers few (or new) details.

For this series of questions, we want you to think about your experiences prior to COVID-19 and what challenges were faced in providing care for underserved populations. We will ask you to respond later on about the specific compounding impact the pandemic may be having on the access to and delivery of care for these patients.

1. Can you tell us about your current practice and the patient population you treat, specifically the estimated make-up, such as:
   - Racial/ethnic demographics
   - Geographic (rural/urban) demographics
   - Sexual/gender minority demographics
   - Non-English speaking
   - Living in poverty
   - Low literacy and/or low health literacy
   - Medically uninsured or underinsured/Medicaid recipients?

2. Can you tell us what influenced you to work in your particular practice setting?
   - *PROBE:* Did you have experience serving underserved patients in other settings, such as a safety net hospital?

3. What has been your experience in providing care for your underserved patients?
   - What do you find the most rewarding or unique about your patients or practice setting?
     - i. *PROBE:* What are facilitators or positive experiences you have had providing care for the underserved population? (i.e., if you were to give advice to a provider who was new to delivering care to underserved populations, what would you share with them?)

4. What challenges do you feel are unique to your setting?
   - *PROBE:* What challenges have you faced in providing care for the underserved?
     - ii. *PROBE:* How do you provide care for underserved populations with complex comorbidities?
     - iii. Do you feel that these patients tend to be more or less engaged in their care? (e.g., less able to self-advocate within the system; have a more paternalistic relationship with the provider and not take an active role in informing their care decisions)
     - iv. What solutions have you employed to overcome these?

5. What are some challenges that you feel your patients experience with their cancer care? (e.g., transportation, childcare, inability to take time off from work)
    i. PROBE: How do socioeconomic status (such as insurance, transportation, housing, income, and education) affect the way that you provide cancer care for underserved patient populations?
    ii. PROBE: Have patients been more challenged to use telemedicine, and if so, what have been the causes? (e.g., broadband, not comfortable with technology, reimbursement)
    iii. PROBE: What approaches have you found to work well in the way that you provide care for patients?
    iv. PROBE: What are some of the approaches you have found to not work so well in the way that you provide care for your patients?
    v. PROBE: Is there a formal department or member of your practice staff that assists these patients?

6. Do you provide care for patients for non-English speakers or patients with limited English proficiency?
    - If yes, how do you approach the care of your patients who have limited English proficiency?
        i. PROBE: Does your practice have access to interpreters? If yes, how are these individuals involved (e.g., in-person, phone, video chat)
        ii. PROBE: What approaches have you found to work well in the way that you care for patients with limited English proficiency?
        iii. PROBE: Are there any other cultural practices that we have not asked about yet that you have noticed influence cancer care?

7. What resources do you currently use to help you educate your patients on the short- and long-term effects of their cancer treatment? (e.g., other members of the care team, community-based partners)
    - PROBE: What other kinds of services or resources do your patients have access to in order to improve their health literacy and understand the cancer treatment process?
    - PROBE: How do these resources and services help or hinder your ability to provide high-quality care to this population?

8. What kinds of formal and informal education/training or experiences have you received in providing care for underserved and/or underrepresented patient populations?
    - PROBE: Can you describe the training in a bit more detail? Did it include training on unconscious bias?
        i. When did you receive the training?
        ii. Who are those trainings offered to? *(e.g., all staff or just providers?)*
        iii. What could be improved upon in those trainings?
        iv. What aspects of the training were helpful for your current practice?
        v. Do you know of other educational materials or training that your peers have received?
    - Probe: Beyond training, what other sources of institutional support do you have to help you address socioeconomic issues that your patients experience? *(e.g., institutional grants, Rx discount programs)*
    - Probe: How do you continue to educate yourself regarding the provision of clinical care (e.g., guidelines)? Do you feel the information available is appropriate to your clinic and to providing care for your underserved patients? (E.g., is guideline-recommended care available/affordable to these patients)
    - Probe: Do you find that others whom you may refer these patients to have adequate training to address their unique needs?

9. What community-based organizations are you aware of, and what role do they play in how you provide cancer care for underserved populations?
    - *PROBE:* How do you coordinate with these organizations (e.g., VNA for home health support)?
    - *PROBE:* Is there an individual within your practice that has been the primary connection to the community resources due to their personal knowledge?
    - *PROBE:* Does your practice utilize a community advisory board? If yes, how do you utilize it?

10. Does your practice use advanced practice practitioners, patient navigators, social workers, or other community health workers to facilitate cancer care for underserved populations?
    - *PROBE:* If so, please describe their roles.

11. ASCO has made addressing clinician burnout a strategic priority. To help understand how the Society may better support you in this space, can you share what the top one or two work-related stressors are that impact your wellbeing?
    - *PROBE:* What physical health consequences have you experienced because of work-related stressors?
    - *PROBE:* How has your emotional wellbeing been impacted due to these stressors?
    - *PROBE:* How do you manage (cope) with challenging work-related situations and events?
    - *PROBE:* What specific signs of burnout have you experienced (e.g., emotional/physical exhaustion, cynicism/depersonalization, feelings of ineffectiveness)?
    - *PROBE:* Has your practice ever had to implement salary or staffing reductions in order to maintain practice viability? If so, how has this affected you?

COVID-19 Question

12. How has COVID-19 affected the care you provide for underserved populations or the patients themselves? *(e.g., financial viability of the practice, patients new to the underserved population, internet access, etc.)*
    - *PROBE:* Has your practice had to implement salary or staffing reductions in order to maintain practice viability?

Wrap-Up

13. What can ASCO do to support you in your efforts to provide quality cancer care for underserved populations and/or support care for underserved people with cancer in general?
    - *Cite specific challenge articulated by key informant brought up previously in discussion to see how they think ASCO could help address this.*
    - Is there anything the Society can do to better support these populations directly?

14. What else is important for us to know that I have not asked?

15. Can we contact you again for any needed clarification or further discussion?

16. As a next step, we may pursue a complimentary patient-facing survey. Can you provide any input as to how a survey like this could be administered to your patient population? What is the best way to hear the patient's experience and perspective?
    - Would you be willing to be contacted to help ASCO conduct a patient-facing survey of this kind?

CONFIDENTIALITY: Thank you for your time; you have been very helpful today. Now that we have completed the interview, I just want to confirm that you *gave / did not give* consent to identify you [by name AND/OR practice/institution] in any written report to follow the analysis of this research. Is that still what you would like to do?

Thank you for your answers and for participating in our study.

Supplemental C. Investigators Qualifications and Backgrounds

Manali I. Patel, MD, MPH, MS—Principal Investigator—is a medical oncologist and health services researcher with an extensive background in medical anthropology and qualitative methods. She uses methods of qualitative

research in all aspects of her studies, including the design and implementation of interventions in which she utilizes community-based participatory research methods. She has led over 25 qualitative studies to evaluate care delivery using mixed-methods research and is the principal investigator on multiple nationally and externally funded studies using mixed-methods research. She has an extensive background in community-based participatory research. She has an appointment as an Associate Professor of Medicine in the Division of Oncology at Stanford University School of Medicine and as a medical services staff oncologist at the Veterans Affairs Palo Alto Health Care System.

Leslie Hinyard, PhD, MSW—Investigator is an epidemiologist and health outcomes researcher with a distinct focus on health disparities and equity in oncology care using real-world evidence. Her recent research emphasis has been on the access to and determinants of palliative care services for patients with metastatic diseases. Dr. Hinyard boasts considerable experience in qualitative study design and analysis and has contributed significantly to over 50 qualitative projects that explore various aspects of healthcare and health outcomes. She serves as the Chair and Associate Professor in the Department of Health Clinical Outcomes Research at Saint Louis University School of Medicine. She also serves as Executive Director at the Advanced HEAlth Data (AHEAD) Institute.

Fay J. Hlubocky, PhD—Investigator is a licensed clinical health psychologist with expertise in psychosocial oncology and a healthcare ethicist at the University of Chicago Medicine in the Department of Medicine, Section of Hematology/Oncology, the Supportive Oncology Program, and the Maclean Center for Clinical Medical Ethics. Dr. Hlubocky received her undergraduate degree from Loyola University Chicago and her Masters of Arts in Healthcare Ethics from Rush University. She completed her Doctor of Philosophy in Clinical Psychology at the Illinois Institute of Technology and her clinical fellowship in psychosocial oncology at the Robert H. Lurie Comprehensive Cancer Center of Northwestern University. Dr. Hlubocky's clinical research and educational efforts center on the impact of the psychosocial aspects in cancer care involving patients, caregivers, and oncologists from the time of diagnosis, treatment, survivorship, and at the end of life. She is an expert in qualitative methodology, collaborates on interdisciplinary research, has published multiple scientific and empirical works and book chapters, and has presented at numerous national and international conferences to promote the significance of addressing patient, caregiver, and oncologist psychosocial needs across the cancer trajectory.

Janette Merrill, MS, CHES—– Investigator—is a senior director in policy programs at the American Society of Clinical Oncology (ASCO), where she provides leadership and managerial oversight of mission strategies related to cancer health equity, prevention, and survivorship. This includes working closely with members to develop and execute strategic projects that include qualitative and quantitative research to meet the Society's long-term goals in these areas. Ms. Merrill earned her bachelor's degree in Speech and Communication Studies, with a minor in Health Science, from Clemson University and a master's degree in health education from Virginia Polytechnic and State University. She has maintained the credential of Certified Health Education Specialist (CHES) since earning it in 2008. She has experience conducting qualitative studies focused on education and curriculum. Ms. Merrill is currently pursuing a Doctor of Health Administration through the Central Michigan University.

Kimberly T. Smith, MSA—Investigator—is an associate director in strategic initiatives at the American Society of Clinical Oncology (ASCO), where she provides programmatic and strategic support to the Society's initiatives related to cancer care delivery and research, with a predominant focus on health disparities and equity. This includes working closely with volunteers to develop instruments for qualitative and quantitative research and execute projects. Ms. Smith has been a team member in ASCO's Policy and Advocacy Department since 2007. She holds a Bachelor of Science in Community Health from the University of Maryland College Park and obtained a Master of Science in Administration with a concentration in public and community health from Trinity Washington University shortly after joining ASCO.

Sailaja Kamaraju, MD, MS—Investigator—is a clinical researcher and oncologist specializing in breast cancer. Dr. Kamaraju's research primarily focuses on health inequities and the development of investigator-initiated trials in cancer. Along with her clinical responsibilities, she leads community-academic partnership projects and outreach efforts in Wisconsin aimed at promoting cancer education and reducing health inequities and has conducted some qualitative research projects. Dr. Kamaraju also holds significant leadership roles through the American Society of Clinical Oncology (ASCO) and the Alliance Foundation. She is also a faculty member in the Division of Hematology-Oncology at Medical College of Wisconsin.

Daniel R Carrizosa, MD, MS—is a Clinical Associate Professor of Hematology and Oncology at the Wake Forest School of Medicine. His primary clinical focus is on head and neck malignancies, thoracic malignancies, and endocrine malignancies. His research encompasses developmental therapeutics, and he focuses on lung screening studies in underrepresented and minority communities. He holds multiple positions at the Levine Cancer Institute as an Associate Program Director for the Hematology and Oncology Fellowship, Medical Director for the Disparities and Outreach Program, and Section Head of the Head and Neck Division. He has limited experience conducting qualitative research.

Tricia Kalwar, MD, MPH—is a hematology and oncology specialist with almost 15 years of extensive experience committed to caring for oncology patients and addressing disparities among underserved populations. Her academic journey includes a master's in public health, emphasizing epidemiology, preceding her medical school graduation in 2006. In this role, she has conducted studies using mixed-methods research. She is the Section Chief of Hematology and Oncology at the Miami VA Healthcare System.

Lola Fashoyin-Aje, MD, MPH—Investigator—is a medical oncologist and Deputy Director in the Division of Oncology 3 (DO3) in the Office of Oncologic Diseases (OOD) at the Center for Drug Evaluation and Research of the Food and Drug Administration (FDA). In this role, she provides clinical, scientific, and regulatory policy guidance and oversight to multidisciplinary teams reviewing drugs and biologics under development for the treatment of solid tumor malignancies. Dr. Fashoyin-Aje is also an Associate Director at the FDA Oncology Center of Excellence, where she leads initiatives to address clinical and regulatory science and policy issues impacting oncology drug development. Prior to joining the FDA, Dr. Fashoyin-Aje completed her undergraduate and graduate training at Columbia University and Yale University, respectively, and received her M.D. degree from the University of Rochester School of Medicine and Dentistry. She has limited qualitative research experience. She completed postgraduate training in internal medicine and medical oncology at Johns Hopkins.

Scarlett L. Gomez, PhD, MPH—Investigator—is a Professor and Vice Chair for Faculty Development in the Department of Epidemiology and Biostatistics and Co-Leader of the Cancer Control Program of the Helen Diller Family Comprehensive Cancer Center at the University of California, San Francisco. She is Director of the Greater Bay Area Cancer Registry, a participant in the NCI SEER (Surveillance, Epidemiology, and End Results) program and in the California Cancer Registry. She has led and collaborated in qualitative research to discern themes underlying cancer disparities and inequities. She has also conducted research that utilizes qualitative findings in developing quantitative survey instruments (mixed methods).

Sanford Jeames, DHA—Investigator—is an adjunct professor at Huston-Tillotson University and a community health educator and patient advocate. His interests include health education, cancer prevention, and healthier lifestyle interventions, with a focus on underserved populations. He served on the Steering Committee for the Men of Color Health Awareness Program in Springfield, Massachusetts, and as Co-Chair for the Prostate Cancer Working Group of the Health Equity and Disparities Workgroup for the Massachusetts Comprehensive Cancer Control Plan. He has some experience conducting qualitative studies focused on improving cancer care for minority populations.

Narjust Florez, MD—Investigator– is a thoracic oncologist at the Dana-Farber Cancer Institute/Harvard Cancer Center and Associate Director of the Cancer Care Equity Program. She specializes in targeted therapy for lung cancer and care for women with lung cancer and leads the Sexual Health Assessment in Women with Lung Cancer (SHAWL) Study. She has conducted five qualitative studies. Dr. Florez also serves as an assistant professor at Harvard Medical School.

Sheetal M. Kircher, MD—Investigator—is an associate professor at Northwestern University, where she conducts clinical work focusing on the care of patients with gastrointestinal malignancies. Her research focuses on improving the quality of cancer care and better understanding the trends in the use and expenditure of services and drugs. She has limited experience conducting qualitative research.

William D. Tap, MD—Investigator—is a medical oncologist focused on providing comprehensive, advanced cancer care in sarcoma and oncology translational medicine. Furthermore, he contributes to the Adolescent and Young Adult Program at Memorial Sloan Kettering to improve holistic care for cancer patients and sufferers of rare disease. His serves as a tenured member at Memorial Sloan Kettering Cancer Center, Professor of Medicine

at Weill Cornell Medical College, Chief of the Sarcoma Medical Oncology Service, and Co-Director of the Lisa and Scott Stuart Center for Adolescent and Young Adult Cancer and the MSKCC Sarcoma Center. He has limited experience conducting qualitative research.
